# Construction Notes

**Published:** 1893-09-23

**Authors:** 


### CONSTRUCTION NOTES.

### Dartford Cottage Hospital.

This hospital has been designed by Mr. Tait, and will accommodate 16 patients, providing two extra rooms for emergency cases, and also apartments for the matron. The site selected is on the East Hill. The plans show a frontage of 100 feet, and a depth of 200 feet. The land can be secured on lease with option of purchase. The total amount needed to be raised is £2,850 to cover the expenses of the building.

### Fever Hospital at Larbert.

This hospital is to be erected on a site between Bannockburn and Larbert, which the Central District Committee of the Stirlingshire County Council have obtained from Sir James Maitland on almost nominal termss. In the meantime the plans show two main wards, each divisible into two by a moveable partition for separation of the sexes. There are also two observation wards, and the total number of beds arranged for at present is ten. The walls of all the buildings are to be of brickwork and faced. The architects are Messrs. M'Luckie and Walker, Stirling.

### Hospital Ship at Ipswich.

This new floating hospital is intended for the reception of cholera cases which may occur within the port and borough. It is designed for the accommodation of six patients in two wards, the dimensions of each ward respectively being 13 feet by 10 feet high, two windows being provided for each. In front of these wards is the caretaker's cabin, and in the rear the kitchen and general administration department, as also the nurses' cabin. There is also a lavatory with patent apparatus, a washing-room, and a strong storage. The material used in the construction is stained pine wood. A promenade runs round the Dock house. The vessel has been three months in hand, and fitting and furnishing are not yet complete; the total cost of her purchase and arrangement into a floating hospital is £700. The contractors for the work are Messrs. Orvie and Fuller.

### Opening of the Nurses' Home, Derbyshire Infirmary.

The first of the various blocks comprising the New Derbyshire Royal Infirmary, was opened at the end of June. It was considered desirable at the commencement of the building to push on this particular work as quickly as possible. The contractors, Messrs. Walker and Slater, are to be congratulated on the speedy progress which they have made. This Nurses' Home is one of the best appointed in the United Kingdom. In architectural design it is imposing, whilst the utmost attention has been paid to every detail in the interior. The primary objects of the architects, Messrs. Young and Hall, of 17, Southampton Street, W.C., was to ensure a thorough system of drainage and sanitation, and in order to effect this the basement or foundation of the building is so constructed that the drains are very easily got at. An excellent flushing tank, capable of holding 150 gallons of water, has been made by Messrs. Slater, of London, who supplied also the heating apparatus. The basement floors are fireproof. On the first floor a long corridor, 8 feet wide, leads to nine bed-rooms, each 13 feet by 10, a box-room, superintendent's and other sitting-rooms, lavatory, and baths. On the second floor are 20 bed-rooms, with a similar number on the third. On the ground floor is the head nurse's sitting-room (22 by 22), also a receiving-room (10 by 12). The probationers'-room is 20 by 13, and there is a room for visitors. The total length of the building is 116 feet. There are large ventilators over all the doors. The bath-rooms are fitted in a very complete manner, and 10 are fitted with hot and cold water. The plumbing work in the interior of the building has been carried out by Messrs. Dent and Elliard, of London. The exterior of the building is of red brick, similar to the rest of the infirmary, the style of which is Elizabethan. The home contains 49 bed-rooms, and is detached from the main building. The estimate for the cost is £7,000.

